# Heart Rate Variability Monitoring during Sleep Based on Capacitively Coupled Textile Electrodes on a Bed

**DOI:** 10.3390/s150511295

**Published:** 2015-05-14

**Authors:** Hong Ji Lee, Su Hwan Hwang, Hee Nam Yoon, Won Kyu Lee, Kwang Suk Park

**Affiliations:** 1 Interdisciplinary Program for Bioengineering, Graduate School, Seoul National University, Seoul 110-799, Korea; E-Mails: hongjidan@bmsil.snu.ac.kr (H.J.L.); lostzoo@bmsil.snu.ac.kr (S.H.H.); hnyoon@bmsil.snu.ac.kr (H.N.Y.); wongyu86@bmsil.snu.ac.kr (W.K.L.); 2 Department of Biomedical Engineering, College of Medicine, Seoul National University, Seoul 110-799, Korea

**Keywords:** capacitively coupled electrodes, conductive textiles, ECG, HRV monitoring

## Abstract

In this study, we developed and tested a capacitively coupled electrocardiogram (ECG) measurement system using conductive textiles on a bed, for long-term healthcare monitoring. The system, which was designed to measure ECG in a bed with no constraints of sleep position and posture, included a foam layer to increase the contact region with the curvature of the body and a cover to ensure durability and easy installation. Nine healthy subjects participated in the experiment during polysomnography (PSG), and the heart rate (HR) coverage and heart rate variability (HRV) parameters were analyzed to evaluate the system. The experimental results showed that the mean of R-peak coverage was 98.0% (95.5%–99.7%), and the normalized errors of HRV time and spectral measures between the Ag/AgCl system and our system ranged from 0.15% to 4.20%. The root mean square errors for inter-beat (RR) intervals and HR were 1.36 ms and 0.09 bpm, respectively. We also showed the potential of our developed system for rapid eye movement (REM) sleep and wake detection as well as for recording of abnormal states.

## 1. Introduction

With the population aging around the world, interest in leading a healthy life rather than undergoing post-sickness treatment has been rising. To realize this, long-term home healthcare systems that can detect abnormal signs in healthy people at an early stage and observe the progress of disease in non-hospitalized patients have been developed. However, taking manual measurements of physiological signals several times a day is inconvenient to people. Therefore, it is important to obtain valuable information on health condition every day without interfering with daily activity.

Unconstrained and unconscious home-based daily health monitoring systems measuring electro-cardiograms (ECGs), ballistocardiograms (BCGs), phonocardiograms (PCGs), and photo-plethysmograms (PPGs) have been developed by various researchers [1,2,3,4,5,6,7,8,9,10,11,12,13,14,15,16,17,18,19,20]. However, such non-contact sensing systems are vulnerable to motion artifacts. Therefore, considering that we spend a third of our lives sleeping and make relatively few movements during this period, the most suitable approach is long-term health monitoring during sleep. This approach would also minimize the intrusion on daily activities and enable accumulation of a large amount of data.

In health monitoring systems, ECG signals are most widely used for observing heart or cardiovascular functions by beat-to-beat analysis because mechanical signals are not as sharp and clear [16]. The conventional ECG measurement systems require wired and wet adhesive Ag/AgCl electrodes, but this is not suitable for daily long-term monitoring because of the use of disposal electrodes, skin irritation from direct contact electrodes, and limitation of activity caused by the wired system. Therefore, several researchers have proposed dry electrodes and non-contact electrodes [21]. However, dry electrodes could lead to metal allergy due to direct contact with bare skin [19]. To overcome these limitations, capacitively coupled electrodes that use non-contact sensing technology have been developed [22,23,24].

Several studies have presented unconstrained ECG measurement systems using dry or capacitive sensing methods in a bed during sleep. Ishijima [13] and Devot *et al.* [14] proposed an ECG monitoring system with textile electrodes located on a foot mat and pillow. However, because this system requires direct contact with the bare skin of the neck and foot, there was a contact problem when sleeping with legs bent or sleeping outside the pillow. In addition, since the textile was attached on the entire pillow, the ECG signals were greatly influenced by noise caused by hair. Park *et al.* [15] presented a system with two electrode sets made of conductive textiles located on the pillow, leg, and shoulders. The ECG signals were measured from the two electrodes in direct contact with skin using a channel selection algorithm. Another approach to measuring ECG with direct skin contact sensors was introduced by Peltokangas *et al.* [16]. Eight embroidered fabric electrodes were sewn on a bed sheet, and ECG was recorded from one contacted bipolar channel among seven channels. These ECG measurement systems using direct skin contact dry electrodes [13,14,15,16] are complicated to set-up and removing because the textile electrodes are attached directly to a mattress or the bedcover and pillowcase. In addition, there is a high risk of contamination due to external exposure of electrodes. Lim *et al.* [17] proposed a non-contact ECG measurement system using an array of eight copper electrodes inserted in a mattress and a large textile electrode. However, because the rigid and hard electrodes protruded from the mattress, the user’s back could become sore when they slept on a bed for a long time. Moreover, the systems developed by Peltokangas *et al.* [16] and Lim *et al.* [17] need a channel selection method because they use multi-channel electrodes, and they might miss better-quality signals in the process of selecting contacted electrodes.

Three groups of researchers designed electrode configurations with wide strips based on conductive textiles for ECG measurement in a bed without direct skin contact [18,19,20]. Wu and Zhang [18] additionally used a leading tail beside each electrode sewn beneath the bed sheet to connect a pre-amp module. However, because the width of electrode (50 cm) covered only the middle part of the mattress, ECG signals could not be recorded when lying on the edge of the bed or on the edge of the electrodes in a lateral posture. Ueno and Yama [19] and Ishida *et al.* [20] proposed a bed-sheet unit, but it had some limitations in set-up and removing the sensors because the rectangular conductive fabric was stuck to the bed sheet with adhesive. In addition, the systems also had a high risk of contamination and a high chance of contacting the electrodes directly with hands, as the textiles were exposed to the external environment.

Regardless of the sensing technology used, the systems mentioned above can provide meaningful information about health, such as acute mental stress, sleep quality, and cardiac arrhythmias (sinus arrhythmia, bradyarrhythmia, Premature Atrial Contractions, Multifocal atrial tachycardia, supraventricular tachycardia, and so on), by analyzing R-peak intervals and heart rate variability (HRV) during sleep [25,26,27,28]. However, there have been very few studies on evaluating the HRV measures in time and frequency domains measured from a capacitively coupled ECG sensing system.

In clinical research, sleep fragmentation due to nocturnal awakenings can lead to daytime sleepiness, obesity, diabetes, cognitive dysfunction, worsening cardiovascular risk, and impairment of immune functions [29,30]. In addition, rapid eye movement (REM) sleep behavior disorder and narcolepsy happen in REM stage [31]. Therefore, it is important to monitor the frequency of nocturnal awakenings and the durations of wakefulness and REM during sleep. The golden standard for sleep monitoring is polysomnography (PSG), but PSG is no guarantee of observing the usual sleep condition because people sleep in an unfamiliar environment with multiple wired sensors attached on the face, head, and limbs. Moreover, there are few clinical centers that install PSG system, and the cost is quite expensive. Therefore, it is a better approach to use unconstrained systems for continuous sleep and cardiovascular monitoring at home.

In this study, we developed a capacitively coupled ECG recording system with conductive textiles for unconstrained daily health monitoring on a bed at home. The main contributions of the proposed system are: (1) ECG was measured without any direct contact between the body and electrodes; (2) the array and size of sensing electrodes were designed to measure ECG with no constraints of sleep posture and location in a bed; (3) polyurethane foam layer was used to minimize the non-contact region caused by body curvature in any posture; (4) our system was covered for durability and easy set-up; (5) the shielding electrode connected to ground was located at the back of two sensing electrodes for external noise reduction; (6) the distance between preamplifier and sensing electrode was shortened to increase signal-to-noise ratio (SNR).

We analyzed the ECG morphology, heart rate (HR) coverage, HRV time and spectral parameters to evaluate the performance of the system. The increased RR intervals by motion artifacts were filled by interpolation. Results of experiments show that the non-contact ECG measurement system is effective to record ECG without any contact to users with high R-peak coverage, low HRV errors, and clear ECG morphology. In addition, we showed that our developed system could detect movement (wake) by increased RR intervals, and also estimate REM sleep using HRV parameters.

## 2. Capacitive ECG Measurement System

**Figure 1:**
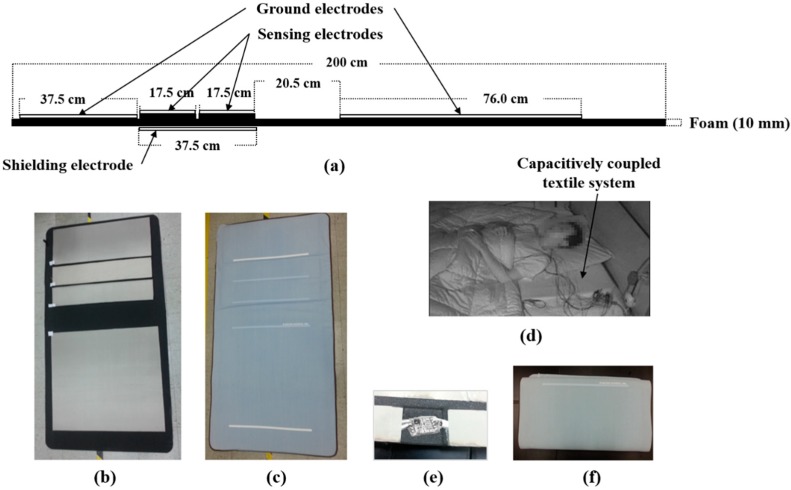
Configuration of capacitively coupled textile system: (**a**) size and array of each electrode in side view; (**b**) actual design in top view; (**c**) system cover; (**d**) experiment installation during PSG; (**e**) preamplifier module; and (**f**) folded system.

We developed the capacitively coupled ECG monitoring system as shown in Figure 1, consisting of sensors for ECG measurement, foam for comfort, and a cover for prevention of pollution.

### 2.1. Conductive Fabric

Conductive fabric (WR-260-PCN, A-Jin Electron, Busan, Korea) was chosen considering its conductivity, durability, weight, elasticity, and sensor suitability, and the entire surface was coated with nickel and copper. The fabric was a halogen-free product that is harmless to humans. The thickness was 0.1 mm, and it had excellent electric conductivity (the sheet resistance < 0.05 Ω/sq). The conductive fabric was used for the sensing electrodes, ground electrodes, and shielding electrode, shown in Figure 1a,b.

### 2.2. Polyurethane Foam

Polyurethane foam was used to provide comfort and to reflect the curvature of the body, especially the waist in a supine posture or the sides in lateral postures. The form had large flexibility and recovery properties, and also able to reduce the air gaps and non-contact region between the textile electrodes and the body. The thickness of the foam was 10 mm, and its density was chosen as 35 kg/m^3^, considering weight and durability. The foam was attached at the back of conductive textile as shown in Figure 1a,b.

### 2.3. System Cover

To avoid contamination of the electrodes by external exposure, a system cover was produced using a 100% cotton fabric, which is general material for bedding. As shown in Figure 1c, five lines on the cover indicate which side contacts with the body and showed where the upper body should lie on, taking into account the different heights of the subjects. For example, upper three lines marked the location of sensing electrodes. Moreover, the backside of the cover had a zipper of length 1.8 m to separate or install the system.

### 2.4. Preamplifier

Preamplifiers were used to increase the SNR. Each preamplifier was coated with a silicone conformal coating (LDC 2577D, Dow Corning Co., Midland, MI, USA) for waterproofing and protection. Because the thickness of the preamplifier was 3 mm (including on-chips), the preamplifier was put into a square hole in the foam next to each sensing electrode to reduce the firm feeling (Figure 1e).

### 2.5. System Design

The system was composed of five conductive textile electrodes, as illustrated in Figure 1a. The front side had two sensing electrodes for ECG measurement (middle) and two ground electrodes for common mode noise reduction (upper and lower). The back side had a shielding electrode to reduce external noise interference, instead of a preamplifier shield made of aluminum plate. Therefore, the shielding electrode was connected to ground and designed to cover the entire two sensing electrodes in the back. All conductive textiles were fixed on the polyurethane foam. By attaching additional foam of thickness 10 mm under each sensing electrode, the area that contacted the body was widened. Therefore, the sensing electrodes were slightly protruded, but the users were not in any discomfort while lying on the system, as the foam was very soft.

The total size of the smart mattress was 100 cm × 200 cm, which is equal to the size of a single mattress. The sizes of the textile electrodes are described in detail in Figure 1a. The size of the upper ground electrode was chosen considering the general pillow size. The configuration of the sensing electrodes was designed as a long strip in order to cover various sleep postures and any location in a bed (17.5 cm × 85 cm). The lower ground electrode was placed under the lower half of the body, and its size was designed to be as large as possible to get stable signals, because the impedance between the body and the electrode was decreased by increasing the contact area (76 cm × 85 cm). Eyelets were used for connecting between the textiles and preamplifier instead of soldering. We also used conductive textiles to protect the wires from external noise interference and to fix them on the foam. The actual installation of the system on a bed is shown in Figure 1d, in which the wires were for PSG. Moreover, the capacitively coupled textile system can be fold for convenient storage like a blanket (Kolon, Seoul, Korea) (Figure 1f).

### 2.6. Hardware Specification

**Figure 2:**
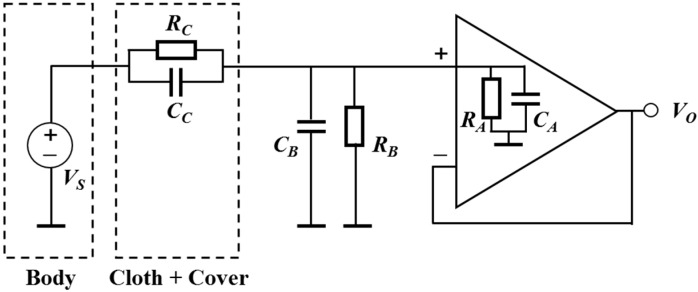
Diagram of the electrical circuit of the electrode.

A high input impedance amplifier is required to measure ECG through clothes. Figure 2 describes the electrical circuit of the electrode. The gain of the circuit is as below:

(1) $$G_{s}(s)=\frac{V_{o}}{V_{s}}=\frac{Z_{B}//Z_{A}}{Z_{C}+Z_{B}//Z_{A}}$$

where *Z_A_* is the input impedance of the operational amplifier (R*_A_*//C*_A_*), *Z_B_* is the impedance of the parallel combination of *C_B_* and *R_B_*, and *Z_C_* is the impedance of the cloth and the cover in this system (R*_C_*//C*_C_*). Since *Z_A_* is sufficiently larger than *Z_B_*, *Z_A_* can be disregarded. Therefore, the gain is defined as:

(2) $$G_{s}(s)=\frac{R_{B}+sC_{C}R_{B}R_{C}}{(R_{B}+R_{C})+s(C_{B}+C_{C})R_{B}R_{C}}$$

**Figure 3:**
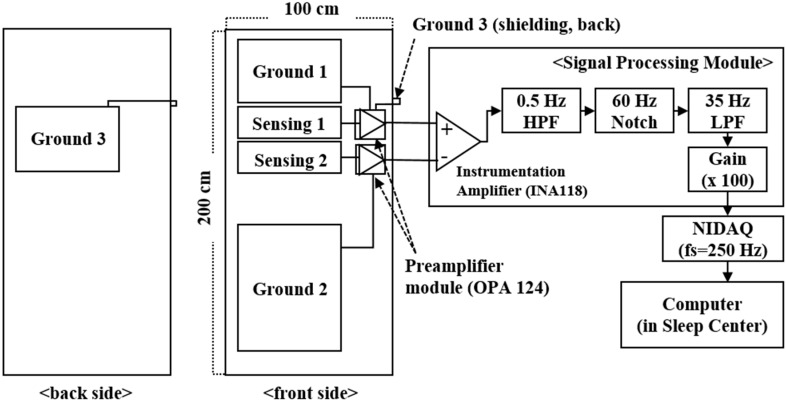
Schematic diagram of the analogue-signal processing module and data acquisition system (fs: sampling frequency).

In our system, an operational amplifier, OPA124 (Texas Instruments, Dallas, TX, USA), which had a high input impedance of 10^13^ Ω || 1 pF was chosen for use as the preamplifier. The hardware module was composed of an amplifier and three FIR Sallen–Key Butterworth filters: a second-order high-pass filter (cutoff frequency, fc = 0.5 Hz), a notch filter (UAF42, Texas Instruments, fc = 60 Hz), and an eight-order low-pass filter (fc = 35 Hz). The total gain was 100 V/V. The overall schematic diagram of the hardware is illustrated in Figure 3.

## 3. Performance Evaluation

### 3.1. ECG Morphology

Before performing the PSG experiment, we verified the signal quality of the developed system by comparing ECG morphology measured from our system to that from the reference system. Seven healthy male subjects (between 23 and 31 years) participated in the experiment. After measuring the ECG for supine posture on the capacitive textile system, the ECG was measured again using reference Ag/AgCl electrodes (Biopac MP 150, Biopac Inc., Goleta, CA, USA) attached on the subject’s back at the similar location in contact with the textile sensing electrodes when the subject lay on our system. The correlations of ECG morphologies measured from the proposed system and reference system were evaluated.

### 3.2. R-Peak Coverages and HRV Parameters during Sleep

#### 3.2.1. Participants

Nine healthy male subjects (between 23 and 31 years) participated in the experiment. Two subjects performed daytime PSG and other seven did nighttime PSG. All subjects gave informed consent to participate, and none of them suffered from a cardiovascular disease or any medical condition.

#### 3.2.2. Protocols

The study was approved by the Institutional Review Board of Seoul National University Hospital (SNUH), and the developed system was installed on a bed in the Sleep Center at SNUH. The subjects slept on the system wearing cotton pyjamas. ECG signals from our system were recorded simultaneously with PSG data using a data acquisition board, NI-DAQ 6221 (National Instruments, Austin, TX, USA), with a 250 Hz sampling rate. The PSG data contained ECG from Lead II (Ag/AgCl electrodes), electroencephalogram (EEG), electrooculogram (EOG), electromyogram (EMG) from the chin, nasal-oral airflow, snoring, and pulse oximetry (SpO2) signals. The PSG data were scored by a polysomnographic technologist and a sleep physician according to the criteria of Rechtschaffen and Kales [32].

#### 3.2.3. Data Analysis

The performance of the proposed system was evaluated by comparing the R-peak coverages from the system with those from Ag/AgCl electrodes (Lead II ECG) during PSG. R-peak coverage was defined as a percentage of the number of R-peak from capacitive textile system of that from Ag/AgCl electrodes on PSG system.

We used an internally developed automatic peak detection algorithm and then checked the detected peaks manually. Moreover, the HRV time and spectral parameters, which are measures of variations in the HR calculated from the RR intervals in the ECG, were assessed using the normalized error (NE) between ECG from Lead II and our system [33,34]. NE is defined as follows:

(3) $$\mathrm{NE}=\frac{\left|X_{\mathrm{Lead}\;\mathrm{II}\;\mathrm{ECG}}-X_{\mathrm{our}\;\mathrm{system}}\right|}{\text{95\%}\;\mathrm{confidence}\;\mathrm{interval}\;\mathrm{length}\;\mathrm{of}\;X_{\mathrm{Lead}\;\mathrm{II}\;\mathrm{ECG}}}\;\times 100(\%)$$

where X is an HRV parameter in time or frequency domain.

The HRV parameters were calculated in 5 min (10 epochs) window with 90% overlap. Since motion artifacts lead to missing R-peak, the increased RR intervals (longer than mean + 3 × SD, where SD is standard deviation) were automatically filled using the Piecewise Cubic Hermite Interpolating Polynomial method. All signal processing was done with MATLAB 2013b.

## 4. Results

### 4.1. ECG Morphology

**Figure 4:**
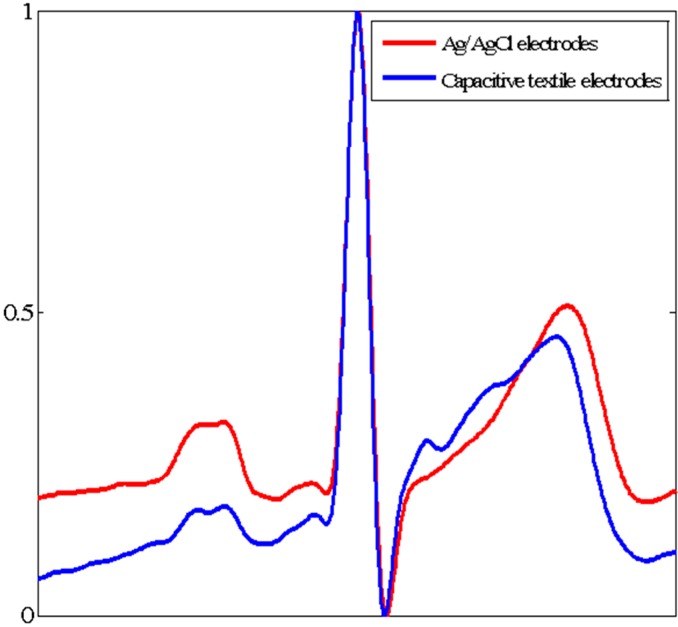
The normalized ECG waveforms measured from Ag/AgCl electrodes (red line) and capacitive textile electrodes (blue line).

The ECG morphologies measured from our developed system and reference Ag/AgCl electrodes were shown in Figure 4. The ECG morphology was filtered with 0.5 Hz–35 Hz. Since the ECGs from both systems were recorded separately, the ECG waveforms in 5 min term had to be averaged to be compared. The QRS complex from Ag/AgCl electrodes (red line) was almost same with that from capacitive textile electrodes (blue line). The mean correlation of ECG morphologies measured from two systems was 0.96.

### 4.2. R-Peak Coverages and HRV Parameters during Sleep

**Figure 5:**
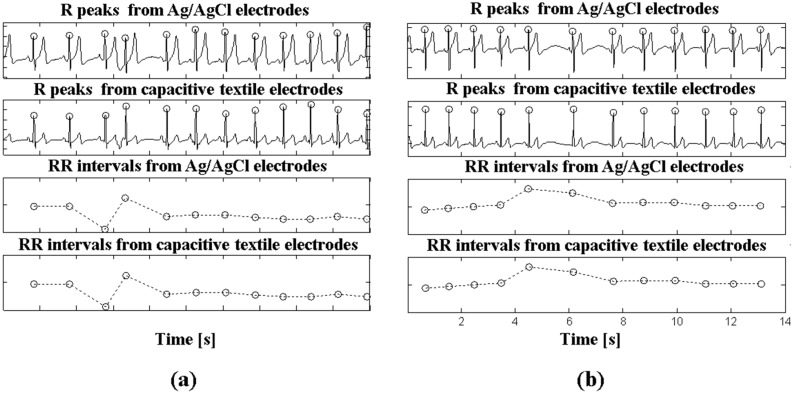
R peaks and RR intervals including (**a**) shorter RR intervals and (**b**) longer RR intervals recorded from Ag/AgCl electrodes (PSG) and capacitive textile electrodes.

Figure 5 illustrates the filtered ECG signals (0.5 Hz–35 Hz) with detected R peaks and RR intervals from the capacitively coupled textile electrodes and Ag/AgCl electrodes (Lead II ECG) during PSG. Although the ECG waveforms were different in both systems because the body parts in contact with the electrodes of each system were different, the developed system reflected the heart beats well even when the RR intervals in a certain period were shorter or longer than normal.

**Figure 6:**
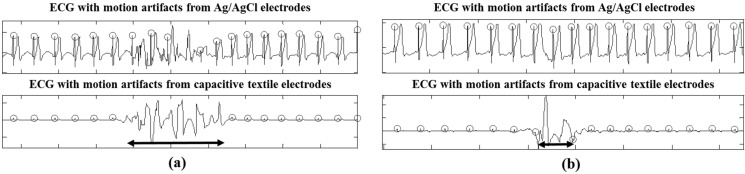
ECG waveforms with motion artifacts due to (**a**) sleep posture change and (**b**) limb movement, from Ag/AgCl electrodes (PSG) and capacitive textile electrodes.

ECG signals with motion artifacts are shown in Figure 6. Large and continuous motion artifacts due to changing sleep posture distorted the ECG signals even in the direct-contact electrodes. However, the ECG signals recorded from the Ag/AgCl electrodes recovered quickly to get R peaks, whereas the ECG data from the capacitive textile electrodes took a longer period before obtaining R peaks in Figure 6a. Small limb movements caused short duration motion artifacts only in the capacitive measurement system, as shown in Figure 6b.

The total recording time, R-peak coverage, and NE of HRV parameters between Ag/AgCl electrode system and capacitive textile system are summarized in Table 1 and Table 2 for the time and frequency domains, respectively. The R peaks coverage ranged from 95.5% to 99.7% of the overall night, with a mean coverage of 98.0%. The smallest mean NE was 0.18% for the mean HR in time domain and 0.15% for the VLF (very low frequency) power in frequency domain. The mean NE of the HRV parameters was less than 5%, with a range of 0.15%–4.20%.

**Table 1:** R-peak coverage and normalized errors of HRV parameters in time domain between Ag/AgCl electrodes and capacitive textile electrodes during PSG for nine subjects.

Subject	Total Time (h.min.s)	R-Peak Coverage (%)	Normalized Errors (%) in Time Domain				
			*SDNN* ^a^	*RMSSD* ^b^	*NN50* ^c^	*pNN50* ^d^	*Mean HR* ^e^
**A**	8.16.09	97.97	1.80	1.80	9.23	9.26	0.08
**B**	8.34.42	99.66	0.45	2.10	0.48	0.48	0.02
**C**	3.51.24	98.04	1.02	6.72	2.40	2.36	0.50
**D**	3.23.47	99.35	0.21	3.78	3.01	3.28	0.05
**E**	8.04.21	99.19	2.26	7.78	0.88	0.95	0.03
**F**	8.21.20	97.41	2.09	5.74	4.58	4.45	0.09
**G**	9.58.45	96.87	0.75	3.18	4.53	4.90	0.11
**H**	8.43.54	95.47	3.54	2.20	1.54	1.77	0.59
**I**	7.56.58	98.06	1.81	4.47	2.45	2.51	0.16
**Mean**		**98.00**	**1.55**	**4.20**	**3.23**	**3.33**	**0.18**

^a^ *SDNN*: Standard deviation of the RR intervals; ^b^ *RMSSD*: Root mean square successive differences of RR intervals; ^c^ *NN50*: Number of pairs of successive RR intervals that differ by more than 50 ms; ^d^ *pNN50*: Proportion of NN50 from the total number of RR intervals; ^e^ *Mean HR*: Mean of heart rate.

**Table 2:** R-peak coverage and normalized errors of HRV parameters in frequency domain between Ag/AgCl electrodes and capacitive textile electrodes during PSG for nine subjects.

Subject	Total Time (h.min.s)	R-Peak Coverage (%)	Normalized Errors (%) in Frequency Domain						
			*LF* ^a^	*HF* ^b^	*LF/HF* ^c^	*nLF* ^d^	*nHF* ^e^	*VLF* ^f^	*nVLF* ^g^
**A**	8.16.09	97.97	0.20	0.26	0.70	0.74	0.74	0.16	1.04
**B**	8.34.42	99.66	0.16	0.38	1.65	1.44	1.44	0.01	0.18
**C**	3.51.24	98.04	1.66	1.38	1.29	1.37	1.37	0.39	5.23
**D**	3.23.47	99.35	0.15	0.48	2.00	1.87	1.88	0.01	0.49
**E**	8.04.21	99.19	0.08	5.84	13.09	15.0	15.1	0.10	5.39
**F**	8.21.20	97.41	0.35	1.08	2.04	2.13	2.13	0.18	1.71
**G**	9.58.45	96.87	0.06	0.20	1.05	1.33	1.34	0.01	0.11
**H**	8.43.54	95.47	0.27	1.16	3.56	2.83	2.84	0.25	0.55
**I**	7.56.58	98.06	0.50	0.95	1.34	1.24	1.24	0.24	5.41
**Mean**		**98.00**	**0.38**	**1.30**	**2.97**	**3.11**	**3.12**	**0.15**	**2.23**

^a^ *LF*: Power in low frequency range (0.04–0.15 Hz).; ^b^ *HF*: Power in high frequency range (0.15–0.4 Hz); ^c^ *LF/HF*: Ratio of LF to HF; ^d^ *nLF*: LF power in normalized units; ^e^ *nHF*: HF power in normalized units; ^f^ *VLF*: Power in very low frequency range (0.0033–0.04 Hz); ^g^ *nVLF*: VLF power in normalized units.

## 5. Discussion

The width of sensing electrodes was designed to be large enough to cover a single mattress, because it is important to record ECG regarding any position on a bed during sleep. However, the increased surface area of sensing electrodes also increased the area where the body does not contact with, and this causes more noise that affect the quality of ECG signals. In addition, Wu and Zhang [18] reported that the amplitudes of QRS complexes acquired from the hands placed inside the electrodes were smaller than from those placed out of the electrode region. To handle these problems, the shielding electrode connected to ground was located at the back of the sensing electrodes, and the distance between the sensing electrode and preamplifier was adjusted as short as possible. Moreover, all the wires from the sensing electrodes and preamplifier were shielded as well to increase the SNR. Therefore, our system was able to record ECG signals with good quality for all subjects no matter what kind of postures and positions they had during sleep.

As shown in Figure 2, the body weight changes the C_B_ in the electrode, because as the body weight increases, the compression on the form between the shielding electrode and the sensing electrode also increases. Despite the fact that the body weight could affect the gain of the system, the reason for using a very soft form instead of a hard and rigid material was to provide comfort and to reflect the curvature of the body for more contact region during sleep. In our experiment, the weights of subjects were ranged from 57 to 85 kg. Making a stable contact environment was more important to measure ECG for long term monitoring because we used the filtered ECG signals.

The ground of the reference system was same with that of our capacitive system, because ECGs were acquired through NIDAQ. Therefore, the ground of PSG system could decrease the noise of the capacitive measurement. However, there was no problem to analyze ECG signals with the increased baseline noise since filtering was used before detecting R-peak. Unlike some other researches on capacitively coupled ECG measurement [19,35,36,37], this study did not use a driven-right-leg circuit, instead of ground, for common mode noise reduction. Since the driven signals give feedback the inverted common-mode voltage to the subject’s body, the signals can affect the EEG measurement from PSG system. Therefore, we used the ground system so that PSG could be measured simultaneously. However, there would be no issue in using a driven-right-leg circuit in our system, which would provide even better performances.

**Figure 7:**
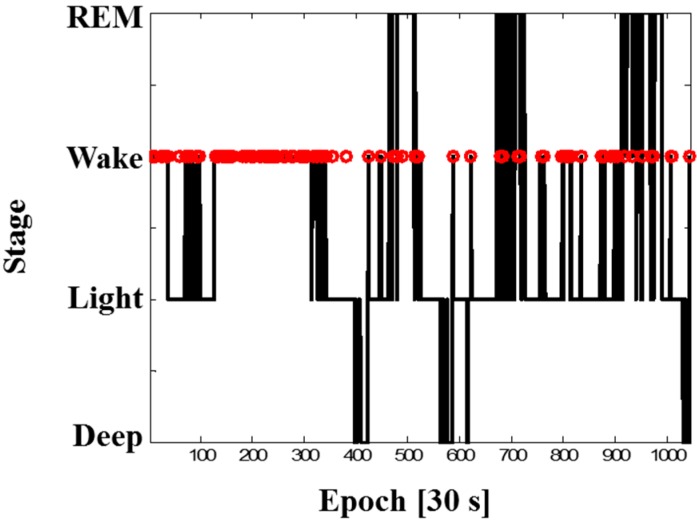
Sleep stages of subject H during PSG and movement (red circles) detected by increased RR intervals measured from the capacitive textile system.

The percentage of R-peak coverage was proportional to the ratio of wake duration to total time (defined as wake ratio) during PSG. For example, subject B had a 2.4% wake ratio during the total time of 8.34 h, and the percentage of R-peak coverage for subject B was 99.7%. On the other hand, subject H had a 27.6% of wake ratio during 8.43 h, and the percentage of R-peak coverage was 95.5%. Figure 7 shows the sleep stages of subject H and his movements (red circles) detected by increased RR intervals (longer than mean + 3 × SD) from the textile electrodes. It can be observed that this subject did not sleep well, because of the unfamiliar environment and many wire sensors. He even sat on the bed for 10 min, tossed and turned a lot during PSG, and then tried to go back to sleep, resulting in a lot of movement in the beginning stage of sleep. Nevertheless, the NEs of HRV measures from subject H were less than 4%, which is under average.

A direct comparison between the performance of our system and the results reported by other studies is difficult because of the use of different datasets, measurement technology, and window size for analyses. Nevertheless, the HR coverage and root mean square errors (RMSEs) of HR are summarized in Table 3 for various technologies and electrode designs.

**Table 3:** Performance summary of related works depending on technologies and electrode design.

Reference	Technologies Used	Electrode Design	Performances	
			*Parameters*	*Results*
Ishijima [13]	Textile, contact	Pillow, foot mat	HR coverage	83%–93%
Devot *et al.* [14]	Textile, contact	Pillow, foot mat	HR coverage	Average 81.89%
Park *et al.* [15]	Textile, contact	Bed, array (multi-channel)	Usable ECG data	Average 84.2%
Peltokangas *et al.* [16]	Textile, contact	Bed, array (multi-channel)	HR coverage	Average 94.9% (85.05%–98.98%)
			RMSE ^a^ for RR interval	4.48 ms
			RMSE of HR	0.27 bpm
			Relative MAE ^b^ of LF, HF, LF/HF	0.89%, 3.90%, 2.20%
Wu and Zhang [18]	Textile, capacitive	Bed, strips	RR interval coverage	98%
			RMSE for RR interval	18.3 ms
			RMSE of HR	1.24 bpm
Our system	Textile, capacitive	Bed, strips	HR coverage	Average 98%
			RMSE for RR interval	1.36 ms
			RMSE of HR	0.09 bpm
			Relative MAE of SDNN, mean HR	0.80%, 0.05%
			Relative MAE of LF, HF, LF/HF	0.29%, 0.93%, 0.88%

^a^ *RMSE*: Root mean square error; ^b^ *MAE*: Mean absolute error.

To compare directly with the existing methods in literature, we also calculated the RMSEs and relative mean absolute error (MAE) between the Ag/AgCl system (PSG) and our developed system. Our system had a high average of HR coverage and a remarkably low RMSEs for RR interval and HR. For HRV parameters, we could only compare with the results obtained from direct-contact methods. Moreover, the relative MAE (%) of our system was considerably lower than those of Peltokangas *et al.* [16].

Our developed system, which is based on a capacitively coupled measurement method, is sensitive to motion artifacts, as shown in Figure 6. The failed R-peak detection usually occurred during sleep posture change or small limb movements. Therefore, since movement usually takes place during wake time, our system has the potential to provide additional information about wake/sleep detection, as in Figure 7 [38].

As our system uses the capacitively coupled method, there are some limitations. First, the material of clothes affects the R_C_ and C_C_ of the electrode that are related to gain as shown in Figure 2. Lee *et al.* concluded that the gain of cotton was higher than that of polyester when using same bias resistor [39]. Second, thick clothes or the blanket should not be caught between the body and the cover. This can happen when changing postures, especially from lateral posture to supine posture. Although some non-electrical methods that do not have this problem have been studied for monitoring heart function, the mechanical signals are more vulnerable to movement and more difficult for beats detection, especially ectopic beats that do not affect the pumping mechanism of the heart [40]. Therefore, unconstrained and contactless ECG recording is the best approach for long-term healthcare monitoring.

**Figure 8:**
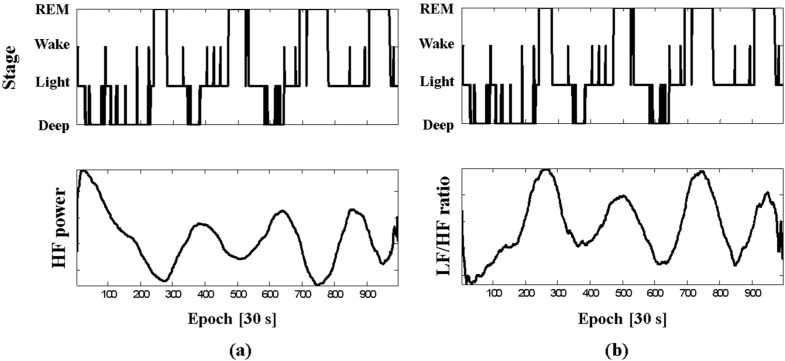
Sleep stages: (**a**) HF power and (**b**) LF/HF ratio from capacitive textile system of subject B during PSG.

As shown in Table 1 and Table 2, the HRV parameters from our system in the time and frequency domains are guaranteed with low errors, especially in mean HR, LF power, HF power, and VLF power. These are useful clinical measures for detecting hypertension, sudden death or cardiac arrest, myocardial infarction, and diabetes [25]. Moreover, the parameters can also provide information on the stress state of the body as the HRV spectral measures are related to changes in the sympathetic and parasympathetic activity of the autonomic nervous system. In addition, the ECG data from our system can be utilized in sleep analyses through the HRV analyses. Figure 8 shows the sleep stages, HF power, and LF/HF ratio from the capacitively measured ECG of subject B during PSG.

These parameters follow the same trend, with HF power decreasing and LF/HF ratio increasing during REM sleep, as reported by sleep-related literatures [14,26]. Therefore, REM and non-REM sleep can be classified using the changes in the spectral powers from our system. Furthermore, as shown in Figure 5, the quality of the ECG signals provided by our system can be adequate for the detection of abnormal states during sleep such as arrhythmia and ectopic beats.

## 6. Conclusions

In this study, we have developed a non-contact ECG measurement system on a bed using conductive textiles for long-term monitoring of heart or cardiovascular function. Our approach was based on designing a more user-friendly system by configuring the array and size of electrodes to measure ECG with no constraints of posture and position in a bed, using a foam layer to provide comfort and follow the curvature of the body, and using a system cover for durability and easy installation. Moreover, we tested the system through HR and HRV analyses to see its potential for monitoring the sleep stages, which is important for observing the physical and psychological states. Our results suggest that the capacitively coupled textile system is suitable for daily healthcare monitoring, with high R-peak coverage, low HRV errors, clear ECG morphology, and accurate R peaks when compared with a commercial system.
